# Biomarkers of hemodynamic stress and aortic stiffness post-STEMI: a cross-sectional analysis

**DOI:** 10.1186/1532-429X-17-S1-P146

**Published:** 2015-02-03

**Authors:** Sebastian J Reinstadler, Hans-Josef Feistritzer, Gert Klug, Luc Huybrechts, Angelika Hammerer-Lercher, Johannes M Mair, Wolfgang-Michael Franz, Bernhard Metzler

**Affiliations:** University Clinic of Internal Medicine III, Cardiology and Angiology, Medical University Innsbruck, Innsbruck, Austria; Central Institute for Medical Laboratory Diagnostics, Medical University of Innsbruck, Innsbruck, Austria

## Background

We aimed to evaluate the association between biomarkers of hemodynamic stress and aortic stiffness assessed at a chronic stage after ST-segment elevation myocardial infarction (STEMI).

## Methods

Fifty-four patients four months after acute STEMI were enrolled in this cross-sectional, single-center study. N-terminal pro B-type natriuretic peptide (NT-proBNP), mid-regional pro-A-type natriuretic peptide (MR-proANP) and mid-regional pro-adrenomedullin (MR-proADM) levels were measured by established assays. Aortic stiffness was assessed by the measurement of pulse wave velocity using velocity-encoding, phase-contrast cardiovascular magnetic resonance imaging.

## Results

NT-proBNP, MR-proANP and MR-proADM concentrations were all correlated with aortic stiffness in univariate analysis (r = 0.378, r = 0.425, r = 0.532, all p < 0.005, respectively). In multiple linear regression analysis, NT-proBNP (ß = 0.316, p = 0.005) and MR-proADM (ß = 0.284, p < 0.020) levels were associated with increased aortic stiffness independently of age, blood pressure and renal function. In receiver operating characteristic analysis, NT-proBNP was the strongest predictor for high aortic stiffness (area under the curve: 0.82, 95% CI 0.67 - 0.96).

## Conclusions

At a chronic stage after STEMI, concentrations of biomarkers for hemodynamic stress, especially NT-proBNP, are positively correlated with aortic stiffness. These biomarkers might also be useful as predictors of high aortic stiffness post-STEMI.

## Funding

Austrian Society of Cardiology.

